# Process options for the recovery of a pentosan-enriched fraction from wheat-based bioethanol thin stillage

**DOI:** 10.1186/s40643-023-00679-8

**Published:** 2023-09-02

**Authors:** Andreas Zimmermann, Marvin Scherzinger, Martin Kaltschmitt

**Affiliations:** Institute of Environmental Technology and Energy Economics, Hamburg University of Technology, Hamburg, Germany

**Keywords:** Stillage, Pentosan determination, Pentosan recovery, Pentosan solubilisation, Distillers’ grains, Ethanol

## Abstract

**Aim:**

Stillage, the main residue from cereal-based bioethanol production, offers a great potential for the recovery of pentosan-type carbohydrates. Therefore, potential process options for the recovery of pentosans from bioethanol thin stillage are investigated and their basic feasibility is demonstrated on a laboratory scale.

**Findings:**

The main result of this work is the development of a three-stage process for pentosan recovery, including solid–liquid separation, pentosan solubilisation and purification. The pentosan content of the thin stillage used here was determined to be about 14% related to dry matter (DM). By means of solid–liquid separation, these pentosans accumulate in the liquid phase (up to 80%), while the remainder (about 20%) is found in the solid phase. Solubilisation of these insoluble pentosans was achieved by using either a hydrothermal, an alkaline or an enzymatic treatment. Here, the results indicate a maximum solubilisation yield of 90% with a hydrothermal treatment using liquid hot water at 180 °C. Ultrafiltration and precipitation are investigated for purification. The most promising process option in this study is solid–liquid separation followed by ultrafiltration. In this case, the total pentosan yield is assessed to be about 48% (based on thin stillage) with a final pentosan concentration of about 30%DM.

**Graphical Abstract:**

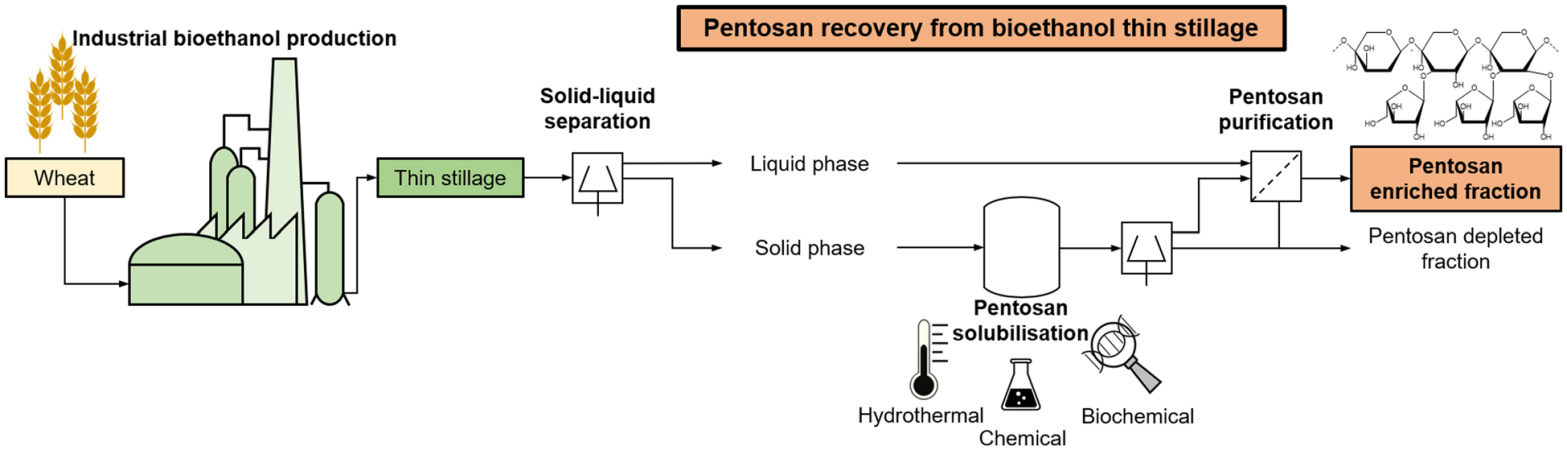

**Supplementary Information:**

The online version contains supplementary material available at 10.1186/s40643-023-00679-8.

## Introduction

Pentosans are a heterogeneous group of oligomeric and polymeric carbohydrates built from monomeric pentoses. They constitute a major fraction of plant cell walls and, as part of hemicellulose, pentosans are typically bound and cross-linked in lignocellulose. In the case of cereal-based pentosans, xylose and arabinose are the main building blocks, hence they are also called arabinoxylans. Wheat contains 2 to 3% and rye even 6 to 8% of pentosans related to the dry matter (DM) of the grain (Belitz et al. 2009). Due to their non-digestibility and their ability to selectively stimulate microbial growth in the intestine of monogastric animals, pentosans can be classified as prebiotics (Roberfroid 2007; Singh et al. 2015). Such prebiotics are of particular interest in the food and beverage sector but also in the feed industry (e.g., for pigs). Currently, fructans and in particular inulin and fructooligosaccharides represent the majority of the prebiotic market. These carbohydrates are so far mainly produced from potential food sources. In contrast, pentosans represent a promising alternative, inter alia, due to their potential recovery from agricultural and industrial residues such as cereal bran (Barros et al. 2022; Misailidis et al. 2009; Singh et al. 2015; Zimmermann et al. 2021).

**Fig. 1:**
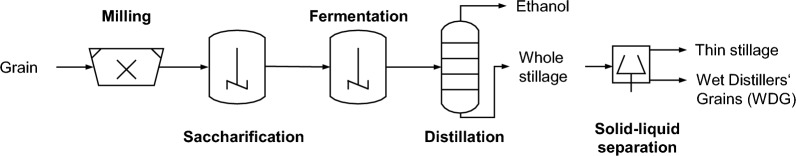
Schematic flow sheet of a conventional bioethanol production

Another potential option for the production of pentosans is their recovery from stillage, the main by-product of bioethanol production processes (Fig. 1) (Alyassin 2019; Chatzifragkou et al. 2015; Flodman et al. 2012; Kosik et al. 2017). In the case of cereal-based ethanol production, such processing typically includes milling and saccharification of the raw cereal followed by alcoholic fermentation. Subsequently, crude ethanol is obtained by distillation and may be further purified in a downstream process. So far, the aqueous residue from the distillation, known as (whole) stillage, is mainly used for low-value applications such as biogas production or as cheap animal feed. By means of solid–liquid separation (e.g., decanter) this whole stillage can be further separated into suspended solids, known as wet distillers’ grains (WDG), and a liquid fraction, so-called thin stillage. Especially the latter is expected to contain soluble carbohydrates, such as monosaccharides and partly pentosans. In addition, there is currently no competitive utilisation path for this thin stillage fraction, although this material stream is recycled in some bioethanol production processes to reduce water consumption. This makes thin stillage particularly interesting for additional valorisation. In comparison, WDG is used directly as fodder for livestock or dried to obtain dried distillers’ grains (with solubles) [DDG(S)] with the advantage of a longer shelf life (Kaltschmitt et al. 2016).

Table 1 shows the pentosan content of stillages from different substrates and stillage fractions after solid–liquid separation indicating increased pentosan contents to be present in the solid fractions. Thus, several processes for the extraction of potentially value-added products from stillage (e.g., pentosans) have been discussed in the literature, mostly focusing on DDGS and/or corn-based stillage so far (Chatzifragkou et al. 2015). However, wheat is the most important raw material for bioethanol production in Germany with a share of about 30% (The Federal Ministry of Food and Agriculture 2021) and the second most used substrate in Europe with about 22% (ePure 2022).

**Table 1:** Composition of different fractions of bioethanol stillage [wet distillers’ grains (WDG); dried distillers’ grains with solubles (DDGS); dry matter (DM); rounded values]

Fraction	Substrate	DM	Pentosans^a^	References
		%	%DM	
Whole stillage	Corn	16	24	Flodman et al. (2012)
	Wheat	22	26	Chatzifragkou and Charalampopoulos (2018)
Thin stillage	Corn	8	4	Kim et al. (2008)
	Wheat	–	5 to 7	Kosik et al. (2017)
	Mixed cereals	11 to 20	–	Lamp (2020)
WDG	Corn	35	21	Chatzifragkou and Charalampopoulos (2018)
	Wheat	–	7 to 11	Kosik et al. (2017)
	Wheat	33	28	Chatzifragkou and Charalampopoulos (2018)
DDGS	Corn	91	12	Pedersen et al. (2014)
	Corn	89	14	Chatzifragkou and Charalampopoulos (2018)
	Wheat	92	13	Pedersen et al. (2014)
	Wheat	–	6 to 9	Kosik et al. (2017)
	Wheat	97	25	Chatzifragkou and Charalampopoulos (2018)

^a^The terms pentosan, hemicellulose and (arabino-)xylan are used as synonyms here

Against this background, it is the aim of this work to systematically investigate the recovery of pentosans from wheat-based bioethanol thin stillage. Therefore, potential process options for pentosan recovery are identified based on analytical results and the state of knowledge. Subsequently, experiments are carried out in order to demonstrate and assess the basic feasibility of such process steps on a laboratory scale.

## Materials and methods

In addition to the analytical methods used here to determine pentosans, this section also describes the systematic approach to a potential pentosan recovery process. It is therefore necessary to anticipate the process developed as a result of this publication.

### Chemicals and sample material

Standards for calibration were obtained from Carl Roth: glucose (anhydrous, ≥ 99%), xylose (≥ 99%) and arabinose (≥ 99%). sulphuric acid (H_2_SO_4_, 96%) for hydrolysis and mobile phase preparation was purchased from Carl Roth as well. Calcium carbonate (CaCO_3_) for neutralisation was purchased from Merck.

The liquid fraction of the stillage, namely thin stillage, was obtained from an industrial bioethanol production plant in East Germany, which processed wheat grain in this production campaign. To enable fractionation by laboratory centrifugation, the thin stillage used had to be diluted with water (1:1 w/w) to reduce viscosity. The dry matter of the raw material was determined based on a gravimetric measurement before and after freeze-drying (Christ, Alpha 1–2 LD). The dried samples were stored tightly sealed at 4 °C for further use and analysis. The liquid fractions were either analysed immediately or stored overnight in a refrigerator at 4 °C for an analysis the next day.

### Pentosan analysis

Pentosan determination was done by high-performance liquid chromatography (HPLC) before and after an acidic hydrolysis step. This means that oligo- and polymeric pentosans are hydrolysed and the released monosaccharides are determined enabling the pentosan amount to be quantified:

- Liquid samples were analysed according to Sluiter et al. (2006) with minor adjustments. Therefore, 10 mL of the liquid sample was transferred into a pressure-tight reaction cell with 16 mm width (Merck). A final concentration of 4.0% (wt.) sulphuric acid (H_2_SO_4_) was adjusted and the sample was hydrolysed at 120 °C for 60 min using a thermoreactor (Merck, TR 420). After hydrolysis, all samples were cooled to room temperature and neutralised with calcium carbonate to a pH of approximately 6 to avoid further degradation reactions. Prior to chromatographic analysis, the neutralised samples were centrifuged (Hettich, Rotixa 50RS) for 20 min with 4950*g* at room temperature. The supernatant was centrifuged again for 30 min with 20,800*g* (Eppendorf, Centrifuge 5414) at 4 °C to obtain a particle-free sample for HPLC analysis.
- Solid samples were also analysed according to Sluiter et al. (2008) with slight modifications. The solid sample was lyophilised, finely ground and passed through a 1-mm sieve. Approximately 0.050 g of this homogenised sample was weighed in a pressure-tight reaction cell (Merck) and 0.350 mL of 72% (wt.) H_2_SO_4_ was added. With the help of a glass rod, the sulphuric acid and the sample were thoroughly mixed and the reaction cell was placed in a water bath at 30 °C for 60 min. The suspension was regularly stirred during this treatment in order to ensure complete wetting. After this first hydrolysis step, 9.8 mL of deionised water was added and the reaction cell was closed. The second hydrolysis step was conducted as before at 120 °C for 60 min in a thermoreactor. The subsequent sample preparation for HPLC analysis was analogous to the procedure for liquid samples (i.e. neutralisation with CaCO_3_ and centrifugation twice to obtain a particle-free sample). The non-hydrolysed samples were centrifuged only and analysed for free monomers.

All samples (before and after hydrolysis) were analysed by an Agilent 1260 Infinity II LC system with a refractive index detector (RID). The separation of monomeric sugars was achieved using an Agilent Hi-Plex H column (7.7 × 300 mm, 8 μm) with the corresponding guard columns. The used method operates at a column temperature of 55 °C using 5 mM H_2_SO_4_ as an eluent with a flow rate of 0.5 mL/min. The injection volume was 20 μL and detection was done by a RID operating at 55 °C.

From this, the pentosan concentration *β*_*p*_ in the hydrolysed sample was calculated according to Eq. (1). The anhydrous factor 132/150 takes into account the water uptake per pentose monomer (molecular weight 150 g/mol) during hydrolysis. *∆β*_*x*_ and *∆β*_*a*_ are the mass concentrations of xylose and arabinose released during pentosan hydrolysis (i.e. the difference between the monomer concentration before and after the hydrolysis step):

1 $$\beta_{p} = \frac{132}{{150}}\left( {\Delta \beta_{x} + \Delta \beta_{a} } \right).$$

Based on the determined pentosan concentration *β*_*p*_, the pentosan content *ω*_*p*_ related to the dry mass of the sample *m*_DM_ can be calculated using Eq. (2). *V* is the corresponding final volume of the analytical hydrolysis:

2 $$\omega_{p} { = }\frac{{\beta_{p} V}}{{m_{{{\text{DM}}}} }}.$$

For each process step *i* and component *j*, the recovery yield *η*_*i,j*_ can be determined related to the total amount of component *j* in the sample prior to the corresponding treatment (index *0*). This figure is calculated according to Eq. (3), in which *m*_DM*,0*_ is the dry mass of the original sample prior to processing and *ω*_*j,0*_ is the respective mass fraction. *β*_*j*_ is the mass concentration of component *j* in the respective liquid volume *V*_*i*_ after processing *i*. The index *j* stands for the compound considered, either xylose (*x*), arabinose (*a*) or pentosan (*p*):

3 $$\eta_{i,j} = \frac{{\beta_{j} V_{i} }}{{m_{{{\text{DM}},0}} \omega_{j,0} }}.$$

In the case of a solubilisation process, the respective yield of pentosan solubilisation *σ*_*p*_ is of interest and can be calculated with Eq. (4). The index *R* indicates the solid residue after a solubilisation step, while the index *0* stands for before solubilisation:

4 $$\sigma_{p} = 1 - \frac{{m_{{{\text{DM}},R}} \omega_{p,R} }}{{m_{{{\text{DM}},0}} \omega_{p,0} }}.$$

### Solid–liquid separation for pentosan fractionation

**Fig. 2:**
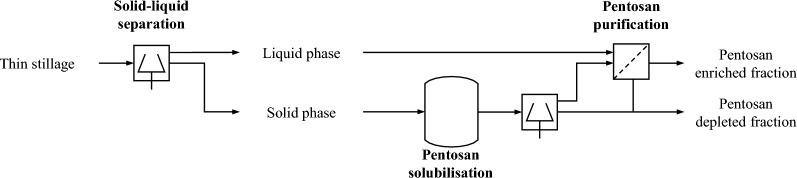
Basic flow diagram of the investigated process for the recovery of pentosans from thin stillage

In order to identify potential process options, the distribution of pentosans over the phases of thin stillage had to be determined first. The aim was to locate the pentosans within the used thin stillage still containing particles (< 1 mm) and thus a solid and a liquid phase. Therefore, diluted thin stillage (1:1 w/w) was fractionated using a laboratory centrifuge (4950*g*, 20 min, 20 °C) and both, the liquid phase (centrate) and the solid phase were analysed for pentosans and their dry matter content. From the knowledge of the resulting pentosan distribution between the two phases (anticipating the results of “Solid-liquid separation for pentosan recovery” section), potential process options can be derived. The derived process investigated here is shown in Fig. 2.

### Pentosan solubilisation

Different types of treatment were used here to extract pentosans from the solid fraction of stillage (Fig. 2). For this purpose, the received thin stillage was first heated to 80 °C to ideally dissolve soluble pentosans. Then, this slurry was allowed to cool to room temperature followed by a centrifugation with 4950*g* for 20 min. Both fractions were collected. The solid fraction was washed with water to remove the residual soluble components. After another centrifugation step, the solid residue was collected, lyophilised and fine ground. This residue was used for the solubilisation experiments with the aim of dissolving out pentosans.

The selection of solubilisation processes was based on literature (Chatzifragkou et al. 2015). This publication summarises different options for the treatment of DDGS of which the following were considered to be promising for the recovery of pentosans from the solid phase of thin stillage:

- Hydrothermal treatment by means of liquid hot water (HT).
- Chemical treatment using alkaline solutions (AT).
- Biochemical treatment by means of enzymes (ET).

An acidic treatment has been excluded as it is usually applied to liberate monosaccharides or partly short-chain oligosaccharides while oligo- and/or polymeric pentosans are wanted here (Roth et al. 2019). Thus, the three different treatments mentioned above were applied to solubilise insoluble pentosans from solids within the stillage using either a one-factor-at-a-time approach or response surface methodology.

#### *Hydrothermal treatment*

Liquid hot water was used in order to dissolve pentosans from the solid stillage residue following the procedure of Kehili et al. (Kehili et al. 2016). The HT was carried out in a 45-mL stainless steel reactor (Berghof, High Pressure Reactor BR-25) with external electric heating jackets. For his purpose, approximately 0.5 g of the ground solid stillage phase was weighed into a polytetrafluoroethylene (PTFE) cartridge. This cartridge was placed in the reactor vessel and 20 mL of water and a magnetic stirrer were added before the reactor was closed pressure-tight. To ensure a liquid water phase even at high temperature, the reactor was pressurised with nitrogen (50 bar). According to current state of knowledge, hemicellulose and thus pentosans are solubilised at about 180 °C (Ruiz et al. 2020). For this reason, the HT was performed at 180 °C for different reaction times (0, 15, 30, 45 and 60 min) each in duplicate. Time recording was started 5 °C below the set temperature (i.e. approximately 5 to 10 min heating time). After the respective treatment time, the reaction was stopped using a water bath for cooling the reactor. For each sample, the reactor content was transferred to a 50-mL centrifuge tube and centrifuged for 20 min at 4950*g*. Both, the supernatant and the residue were analysed for their pentosan content as described in “Pentosan analysis” section.

#### *Alkaline treatment*

Sodium hydroxide solutions (NaOH) with varying concentrations were used for a chemical treatment of the solid stillage phase. Therefore, 0.5 g dried sample was weighed into a 50 mL DURAN® flask (Schott) and 20 mL of the respective NaOH solution was added. Subsequently, the flask was closed and placed in a preheated dry bath (2mag AG) with stirring function (150 min^−1^). The alkaline sample suspension was allowed to warm up for 5 min before the start of the actual reaction time. By means of design of experiment (DoE), the reaction time, the reaction temperature, and the concentration of NaOH were varied using an advanced central composite design (CCD) and the software DesignExpert® (Stat-Ease). The basics of DoE have already been fully described (Pereira et al. 2021). Accordingly, a CCD uses centre points and star points besides the cube points of a design space (Table 2). Based on this, the influence of each factor (here: temperature, reaction time, and NaOH concentration) on a respective response variable (here: pentosan content *ω*_*p*_ and yield *σ*_*p*_) can be estimated. The values of each factor and their corresponding levels are shown in Table 2 (the full data set is included in Additional file 1). The range of these values was chosen based on preliminary experiments and literature values (Flodman et al. 2012).

**Table 2:** Central composite design (CCD) for alkaline treatment (AT) of stillage: values and corresponding levels of factors as well as the basic layout of a three-factor CCD

Factor	Levels					
	− *α*	− 1	0	+ 1	+ *α*	
Reaction time *t*_*R*_ (min)	26	40	60	80	94	
Temperature T (°C)	19	60	120	180	221	
NaOH concentration *c* (mol/L)	0.07	0.10	0.15	0.20	0.23	

#### *Enzymatic treatment*

A biochemical treatment was carried out using 50 mL DURAN® flasks (Schott) and the commercial xylanase solution ROHALASE® VISCO-SEP from AB Enzymes GmbH. The optimum conditions (temperature and pH value) for this ET were determined in preliminary tests using DoE (Additional file 1) (Pereira et al. 2021). For the ET, 0.5 g dried sample was weighed in and 20 mL of McIlvaine buffer solution [citrate–phosphate buffer with pH 4.8 (McIlvaine 1921)] was added. Immediately before the start of the reaction, 20 μL of enzyme solution was added and the closed flask was put into a preheated stirred dry bath (2mag AG) with a stirring speed of 150 min^−1^ at 44 °C. After 5 min of preheating, the reaction time was varied (0, 10, 20, 30, 60, 120 and 1200 min) to examine the pentosan solubilisation at the activity optimum of ROHALASE® VISCO-SEP. Each experiment was conducted in duplicate.

### Downstream processing for pentosan purification

Following Fig. 2, two methods for the enrichment of pentosans were investigated. The starting material is the liquid phase after centrifugation (Fig. 2).

#### *Ultrafiltration for pentosan purification*

Pentosan purification or rather an enrichment of the pentosans can be achieved by means of ultrafiltration (Swennen et al. 2005). For this reason, three stirred ultrafiltration cells (Amicon, Model 8400) with 400 mL each were used in parallel. Flat sheet membranes were obtained from Alfa Laval AB with molecular weight cut-offs (MWCO) of 5, 10 and 20 kDa (UF-pHt™ series). Each cell was filled with 200 mL substrate (liquid phase of thin stillage) and 100 mL of permeate and 100 mL of retentate were collected (i.e. the concentration factor is 2, expressing the initial volume divided by the end volume). The stirring speed was set to 150 min^−1^ and nitrogen was used to pressurise each filtration cell with 4 bar. The permeate and retentate were lyophilised for pentosan analysis (“Pentosan analysis” section) and the dry matter was determined gravimetrically.

#### *Precipitation for pentosan purification*

Another option for pentosan purification and recovery is precipitation by means of ethanol (Swennen et al. 2005). Therefore, the liquid phase of the thin stillage was mixed with ethanol in a 50-mL centrifuge tube to give a final ethanol concentration of 0, 20, 40, 60 and 80%(vol.). The tubes were sealed and mixed in an overhead shaker at room temperature for 30 min. Subsequently, the tubes were centrifuged (4950*g*, 20 min) and both resulting fractions were dried at 50 °C to evaporate ethanol prior to freeze-drying. Each fraction was then analysed for its pentosan content according to the method described in “Pentosan analysis” section.

## Results and discussion

The results are presented and discussed step by step along the process flow (Fig. 2).

### Solid–liquid separation for pentosan recovery

The results of the thin stillage dilution and subsequent solid–liquid separation are shown in Table 3. It shows the dry matter content to be reduced by half through dilution. In contrast, the pentosan content in the diluted thin stillage increases slightly from about 14 to 14.4%DM. As a result of the subsequent centrifugation, the pentosans accumulate in the liquid phase of the thin stillage accounting for about 80% of the total pentosans. Simultaneously, about half of the dry matter and thus other substances end up in this liquid fraction. The wet solid residue after centrifugation comprises about 20% of the total thin stillage’s pentosans and accounts for about 50% of the total dry matter.

**Table 3:** Distribution of pentosans between the different fractions of used thin stillage after solid–liquid separation (centrifugation with 4950*g* for 20 min; mean values of triplicates with standard deviation)

Fraction	Dry matter (DM)^a^	Pentosan content	Share of DM	Share of pentosans
	%	%DM	%	%
Thin stillage	16.77 ± 0.03	14.02 ± 0.06	100	100
Diluted thin stillage (1:1 w/w)	8.37 ± 0.08	14.39 ± 0.08	100	100
Liquid phase of thin stillage after centrifugation	5.36 ± 0.36	21.35 ± 0.40	49.2 ± 0.9	78.4 ± 4.6
Solid phase of thin stillage after centrifugation	22.49 ± 0.10	5.78 ± 0.12	52.9 ± 0.5	21.7 ± 0.6

^a^Related to the fresh mass

Compared to the initial substrate wheat grain [pentosan content about 7.6%DM (*n* = 3)], the pentosan content *ω*_*p*_ increases significantly [up to about 14%DM (*n* = 3)] during bioethanol production. This pentosan enrichment (almost a doubling) is due to the degradation of in particular starch during alcoholic fermentation. This has already been documented (Kosik et al. 2017). In comparison to the literature (Table 1), the pentosan content of the investigated thin stillage is high (e.g., about 6%DM (Kosik et al. 2017)). In the corresponding publication, the thin stillage fraction was obtained from a laboratory-scale production instead of an industrial scale and thus differences are to be expected (e.g., due to recycle streams within the process). A comparison of the fractionated thin stillage (i.e. liquid and solid phase) with the literature is not possible due to a lack of literature.

**Fig. 3:**
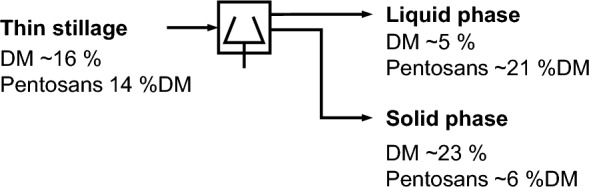
Process step 1: solid–liquid separation of diluted thin stillage [about 8% dry matter (DM)]; (dilution with water 1:1 w/w) using centrifugation

In summary, the majority of the stillage’s pentosans accumulate in the liquid fraction. Thus, such a solid–liquid separation is assessed to be a useful first step for pentosan recovery (Fig. 3). Simultaneously, about one-fifth of the pentosans are present in the stillage’s solid phase. In order to separate these pentosans, they first have to be released. This requires solubilisation. For this reason, a solubilisation step is considered next.

### Solubilisation of pentosans from stillage’s solid phase

The results of the three methods investigated for the solubilisation of pentosans are presented and discussed below. The chapter concludes with a comparison of the solubilisation methods.

#### *Hydrothermal treatment*

**Fig. 4:**
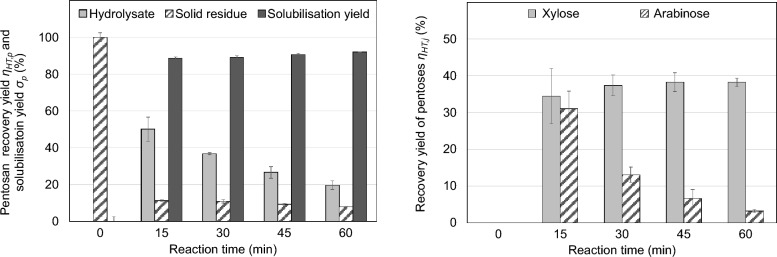
Hydrothermal treatment (HT) of the solid phase of thin stillage for different reaction times at 180 °C: pentosan recovery yield *η*_HT*,p*_ related to total amount of pentosans in the liquid hydrolysate and in the solid residue as well as the pentosan solubilisation yield *σ*_*p*_ (left). Yield of released monomeric pentose *η*_HT*,j*_ related to the total amount of pentose *j* in the solid (right) (mean values of duplicates; error bars correspond to the standard deviation)

Figure 4 shows the results of a hydrothermal treatment (HT) of thin stillage’s solids applying 180 °C and 50 bar for different reaction times.

As shown, the pentosan recovery yield *η*_HT*,p*_ in the resulting hydrolysate initially increases and then starts to decrease with increasing reaction time [Fig. 4 (left) light grey bars]. Simultaneously, the pentosan yield in the solid (crosshatched bars) decreases continuously over the reaction time and so, conversely, the pentosan solubilisation yield *σ*_*p*_ increases (dark grey bars). These results indicate that pentosans are solubilised but degraded at the same time under these harsh conditions. The highest yield of pentosan solubilisation *σ*_*p*_ is achieved at the longest reaction time (about 90%). However, the pentosan recovery yield in the hydrolysate (at this point) is quite low (about 20%) due to degradation reactions, i.e. the degradation of pentosans to pentoses and derivatives such as furfural. Consequently, there must be a trade-off and a respective optimisation is required to maximise both the yield of solubilisation and the amount of pentosans in the hydrolysate (here expressed by the recovery yield *η*_HT*,p*_).

Figure 4(right) shows the corresponding course of the monomeric pentoses *η*_HT*,j*_ released during HT indicating that both monomers are solubilised as well. While the yield of released xylose (light grey bars) increases with increasing reaction time, the yield of arabinose (crosshatched bars) decreases successively. This confirms the degradation of pentosans to xylose and arabinose in parallel to their solubilisation. It also shows the further degradation of these monomers, in particular to furfural and derived products (data not shown). These consecutive reactions explain the decrease of monomeric pentoses over the reaction time and occur especially for arabinose. This demonstrates the lower stability of arabinose compared to xylose (under the given conditions) and has already been described for hydrothermal treatments (Zerback et al. 2022).

#### *Alkaline treatment*

**Fig. 5:**
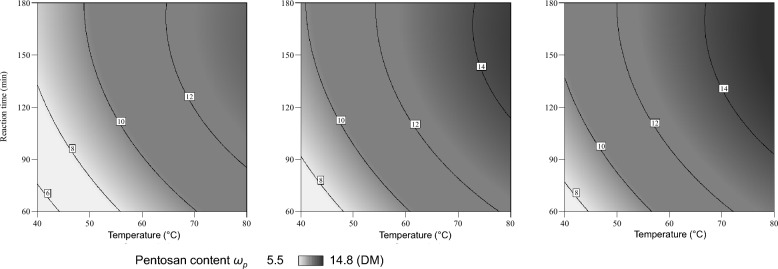
Alkaline treatment (AT) with sodium hydroxide (NaOH) using design of experiment: influence of temperature, reaction time and concentration of NaOH on the pentosan content *ω*_*p*_ in (%DM) of the solid residue for 0.10 mol/L NaOH (left), 0.15 mol/L NaOH (middle) and 0.20 mol/L NaOH (right)

**Fig. 6:**
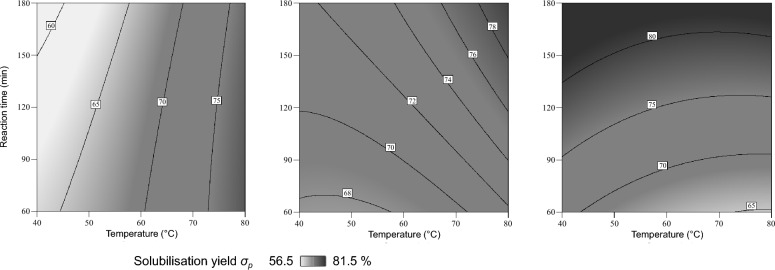
Alkaline treatment (AT) with sodium hydroxide (NaOH) using design of experiment: influence of temperature, reaction time and concentration of NaOH on the pentosan solubilisation yield *σ*_*p*_ in (%) for 0.10 mol/L NaOH (left), 0.15 mol/L NaOH (middle) and 0.20 mol/L NaOH (right)

Figures 5 and 6 show the results of the alkaline treatment (AT) with NaOH according to the experimental design (Table 2) in contour plots.

The influence of temperature, reaction time and NaOH concentration on the pentosan content *ω*_*p*_ in the solid residue after AT is comparatively simple (Fig. 5). The higher the severity (i.e. higher temperature, longer reaction time, higher NaOH concentration), the higher the pentosan content in the corresponding solid residue. In other words, the higher the severity, the apparently less pentosans are solubilised.

The influence of temperature, reaction time and NaOH concentration on the pentosan solubilisation yield *σ*_*p*_ is found to be more complex (Fig. 6). For low NaOH concentrations, the solubilisation yield increases with increasing temperature, but the influence of the reaction time is negligible. In contrast, for high NaOH concentrations, the solubilisation yield is found to be more dependent on reaction time than on temperature. This shows that pentosans are solubilised as a result of the AT.

Considering also Fig. 5 (i.e. an increasing pentosan content in the solid phase with increasing severity), it can be concluded that other substances are solubilised as well. If this happens in larger scale than for pentosans, their apparent accumulation in the solid phase may occur (as in this case). The literature indicates that proteins are also solubilised under these conditions (Lamp 2020). With a view to the subsequent purification, the yield of pentosan solubilisation should be as high as possible. At the same time, the pentosan content in the solid residue should be as low as possible (indicating a comparatively selective pentosan solubilisation). For these reasons, the AT conditions are considered to be most suitable at low pentosan contents (in the solid) and concurrently high pentosan solubilisation yields (in the liquid). This trade-off can be analysed using the results of design of experiment (DoE) with the Software DesignExpert®. As a possible result of this numerical optimisation, AT conditions of 40 °C, 0.18 M NaOH and 180 min were found to be most suitable. According to the calculation, a pentosan solubilisation yield *σ*_*p*_ of about 80% can be achieved under these AT conditions (resulting in a solid residue with a pentosan content *ω*_*p*_ of approximately 10%DM). According to the underlying models (of the DoE), other factor combinations may give comparable results (i.e. pentosan solubilisation yields). However, in this case it was tried to keep temperature and NaOH concentration minimal (i.e. from a process engineering point of view a low energy demand and a low chemical demand) while the pentosan solubilisation yield should be maximised and the pentosan content in the remaining solid should be minimised. The analysis of variance (ANOVA) for both models and the corresponding data can be found in Additional file 1.

The AT conditions determined are consistent with literature reporting comparable conditions for alkaline treatment. For example, for cereal bran, treatment conditions of 40 °C, 0.2 M NaOH and 120 min are reported (Alyassin 2019). For dried distillers’ grains (DDG), a treatment of 50 °C and 0.77 M NaOH for 180 min is used for extraction (Anderson and Simsek 2019).

#### *Enzymatic treatment*

**Fig. 7:**
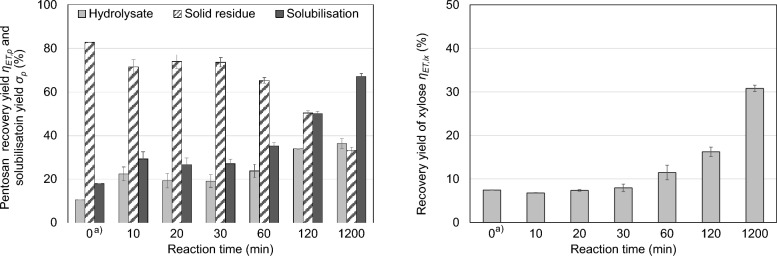
Enzymatic treatment (ET) of thin stillage’s solids with ROHALASE® VISCO-SEP at 44 °C and pH 4.8 (buffer): pentosan recovery yield *η*_ET*,p*_ related to total amount of pentosans in the liquid hydrolysate and in the solid residue as well the pentosan solubilisation yield *σ*_*p*_ (left). Recovery yield of released xylose *η*_ET*,x*_ (%) related to the total amount of xylose in the solid (right) (mean values of duplicates; error bars correspond to the standard deviation). ^a)^ *t*_*R*_ = 0 means a treatment at 44 °C and pH 4.8 (buffer) for 30 min without enzymes

Figure 7 shows the results for an enzymatic treatment (ET) of the thin stillage’s solid phase in comparison to a non-enzymatically treated sample (here: *t*_*R*_ = 0 meaning an extraction using only a buffer with pH 4.8 at 44 °C for 30 min without enzymes).

The pentosan yield *η*_ET*,p*_ in the solid residue decreases already after a short reaction time of *t*_*R*_ = 10 min, while the yield in the hydrolysate increases [Fig. 7(left) crosshatched bars]. This means that the enzymes (not just the buffer) significantly solubilise pentosans from the solid phase. However, even longer reaction times (up to 60 min) do not significantly influence the recovery yield in the liquid phase. After a reaction time of 60 min the pentosan content in the solid decreases more significantly. Vice versa the pentosan content in the hydrolysate (light grey bars) increases with the reaction time. In the same way, the pentosan solubilisation yield *σ*_*p*_ increases with time (dark grey bars). As with the other treatment methods, a trade-off must be made between maximum solubilisation and the resulting pentosan yield (i.e. pentosans actually present in solution after enzymatic treatment). The ET performed resulted in up to 67% pentosan solubilisation yield *σ*_*p*_ after 20 h. The pentosan content *ω*_*p*_ of the resulting hydrolysate is estimated to be 20.7 ± 0.3%D) (*n* = 2) with a pentosan recovery yield *η*_*p*_ of about 35% in the hydrolysate. Consequently, other substances are dissolved as well, presumably due to the buffer as the enzymes are highly selective.

Figure 7(right) shows the course of the xylose released as function of reaction time. Initially, there is no detectable effect of the ET. From a reaction time *t*_*R*_ ≥ 60 min xylose is released significantly as a consequence of the ET. This means, not only pentosans but also pentosan-derived xylose monomers are solubilised. These xylose monomers are the result of an enzymatic hydrolysis of the pentosans and explain the decrease in pentosan yield [Fig. 7 (left) light grey bars]. In contrast to the hydrothermal treatment (HT), no further degradation of the released xylose monomers (e.g., to furfural) was observed.

In summary, the ET results in a reduction of the pentosan content in the solid, a solubilisation of the pentosans and a simultaneous release of xylose from the pentosans. Whether these pentosans are first dissolved (prior to enzymatic hydrolysis) or hydrolysed by the enzymes directly, cannot be determined here. The pentosan content in the dry substance of the resulting liquid phase (hydrolysate) is higher than in the case of hydrothermal and also alkaline treatment, at approximately 20%DM. Nevertheless, accompanying substances are dissolved, which can possibly be prevented by the use of enzymes without buffer. In this way, a comparably pure extract in terms of pentosans should be obtained, which simplifies the subsequent purification and is therefore preferable.

#### *Interim conclusion*

**Fig. 8:**
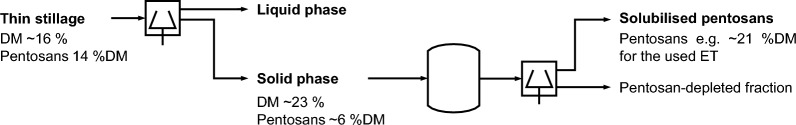
Process step 2: pentosan solubilisation after solid–liquid separation of diluted thin stillage [dry matter (DM), enzymatic treatment (ET)]

All solubilisation processes applied here have in common, that pentosan degradation occurs resulting in a reduction of the polymeric pentosan content in the liquid phase (over the treatment time). In comparison, hydrothermal treatment using liquid hot water has achieved the best results regarding the pentosan solubilisation yield *σ*_*p*_ (up to 90%) but shows significant pentosan degradation. When it comes to the pentosan content (i.e. purity) in the resulting product phase or rather hydrolysate, enzyme use is assessed to be more appropriate. Here, a solubilisation yield *σ*_*p*_ of up to almost 70% was achieved while the resulting liquid phase has a pentosan content of approximately 21%DM probably facilitating purification. Against this background, pentosan solubilisation is assessed as a potential second step in a pentosan recovery process (Fig. 8).

Next, further downstream processing (i.e. enrichment of pentosans) is addressed and considered as a potential third step for pentosan recovery from thin stillage.

### Downstream processing of stillage’s liquid phase

For downstream processing of the liquid phase, ultrafiltration and precipitation with ethanol are typically used (Alyassin 2019). Both methods have been investigated for the liquid phase of thin stillage (Fig. 2). The corresponding results are presented and discussed below.

#### *Ultrafiltration*

**Fig. 9:**
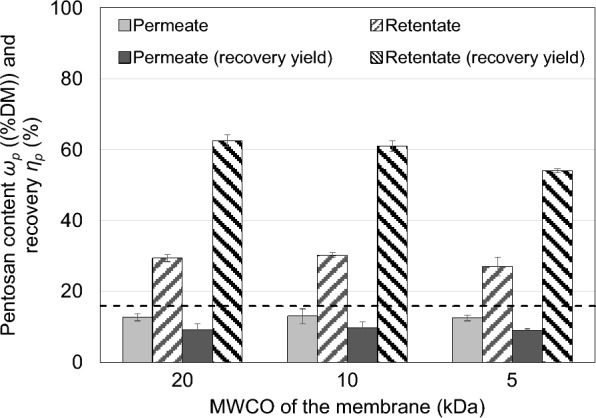
Ultrafiltration of the liquid phase of thin stillage with different molecular weight cut-offs (MWCO): pentosan content *ω*_*p*_ of the permeate and retentate and the recovery yield of pentosans *η*_*p*_ for the retentate related to total amount of pentosans prior to ultrafiltration (mean values of triplicates; error bars correspond to the standard deviation; the dotted line indicates the pentosan content of thin stillage)

Ultrafiltration was performed using the liquid supernatant of thin stillage after centrifugation. The results are shown in Fig. 9. In comparison to the feed (dotted line), the pentosan content decreases in the permeate (light grey bars) while pentosans accumulate in the retentate (crosshatched bars). This means, pentosans are predominantly retained by these membranes. However, a change in the membranes’ molecular weight cut-off (MWCO) (within the range investigated) does not significantly influence the pentosan contents in the permeate and retentate. This is also true for the dry matter of both phases which are also not significantly influenced by the MWCO of the investigated membranes (data not shown). The pentosan recovery *η*_*p*_ indicates that most of the pentosans are retained by the membranes (hatched bars). Thus, these pentosans have high molecular weights (≥ 20 kDa) or are not truly dissolved but still bound to small particles. The maximum pentosan content *ω*_*p*_ in the retentate (about 30%DM) is achieved with a 10-kDa membrane and about 61% recovery in comparison to the amount of pentosans prior to this purification step. Using the 10-kDa membrane results in an overall recovery yield of about 48% from the original thin stillage to the retentate and a doubling of the purity.

The ultrafiltration was performed in batch operation using a stirred dead-end filtration leading to sedimentation and probably the formation of a secondary membrane on/within the actual ultrafiltration membrane. As these sediments adhered to the membrane, it was not possible to fully recover the retentate and the respective pentosans. These losses explain the gap in the mass balance (Fig. 9; light grey and hatched bars) and indicate that the actual pentosan recovery yield *η*_*p*_ in the retentate is even higher (up to 90%).

#### *Ethanol precipitation*

**Fig. 10:**
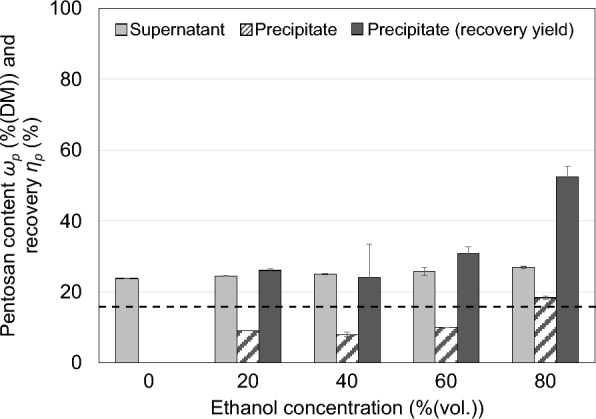
Precipitation of the liquid phase of thin stillage using different ethanol concentrations: pentosan content *ω*_*p*_ in the supernatant and precipitate and the pentosan recovery yield *η*_*p*_ for the precipitate related to total amount of pentosans (mean values of duplicates; error bars correspond to the standard deviation; the dotted line indicates the pentosan content of thin stillage)

Precipitation was performed using different ethanol concentrations. The results are presented in Fig. 10 showing that the pentosan recovery yield *η*_*p*_ (dark grey bars) increases with increasing ethanol concentration and so does the pentosan content *ω*_*p*_ in the precipitate (crosshatched bars). On the other hand, the pentosan content of the resulting supernatant (light grey bars), hardly changes at different ethanol concentrations. The highest recovery in the precipitate is achieved with 80%(vol.) ethanol yielding 52% related to the total amount of pentosans prior to the precipitation step. This precipitation product has a low purity of about 18%DM pentosans due to additionally precipitated substances. The overall recovery yield here is about 41% from the thin stillage to the precipitate with a 20% increase of pentosan purity.

These results are in line with the literature as short-chain carbohydrates and especially monosaccharides remain dissolved even at high ethanol concentrations (Alyassin 2019). In contrast, long-chain carbohydrates such as pentosans tend to precipitate as their solubility decreases in reduced polar media. However, accompanying substances (e.g., proteins) are partly precipitated as well with increasing ethanol concentrations (Lamp 2020). This may explain why the share of pentosans in the supernatant does not changes with increasing ethanol concentration. Since other substances are co-precipitated, the (relative) pentosan content does not change.

#### *Interim conclusion*

**Fig. 11:**
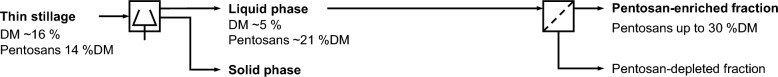
Process step 3: ultrafiltration after solid–liquid separation of thin stillage [dry matter (DM)]

It can be seen that the applied ultrafiltration gives the better results in terms of purity and total yield compared to the applied ethanol precipitation. By means of ultrafiltration a pentosan-enriched fraction with 30%DM could be obtained. Since this is the retentate, a concentration and thus reduction of the water content is usually simple and facilitates further processing. The third step for pentosan recovery from thin stillage is therefore ultrafiltration (Fig. 11). Additional purification effort is required, if the pentosan content of this product is supposed to be increased further.

## Conclusion

**Fig. 12:**
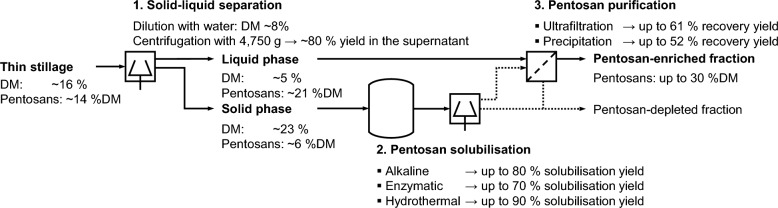
Overview of the developed three-stage process for the recovery of pentosans from thin stillage [dotted arrows indicate potential, not experimentally studied pathways; dry matter (DM)]

The recovery of pentosans from wheat-based thin stillage was systematically investigated and found to be principally feasible using different basic process operations. Based thereon, a respective process was developed and different process options were compared experimentally. Figure 12 shows the developed three-stage process and gives an overview of the corresponding results for the investigated process steps.

The results indicate that solid–liquid separation by means of centrifugation leads to an accumulation of pentosans in the liquid supernatant with 80% recovery yield *η*_*p*_. Subsequent purification results in a pentosan-enriched fraction with up to 30%DM pentosans and 48% overall recovery yield using ultrafiltration with a 10 kDa membrane. This represents the most promising process option investigated here.

In parallel, the solubilisation of pentosans from the residual solid phase (20% of the thin stillage’s pentosans) was investigated. Here, a solubilisation yield *σ*_*p*_ of up to 90% was found using hydrothermal treatment. However, due to the significant solubilisation of other substances (e.g., proteins), the subsequent purification is assessed to be impeded. Concerning this matter, enzymatic pentosan solubilisation offers advantages, as the solubilised pentosans in the liquid phase are comparably pure (about 21%DM). Subsequent purification after pentosan solubilisation was not investigated here, however, ultrafiltration and ethanol precipitation are considered to be promising options as well.

As this process uses well-understood basic process operations, its potential scale-up is assessed to be comparatively easy. Consequently, the developed process for pentosan recovery from thin stillage could be the starting point for additional purification efforts and respective research work to further increase the pentosan content of the target fraction.

### Supplementary Information

**Additional file 1.** Additional material - Process options for the recovery of a pentosan-enriched fraction from wheat-based bioethanol thin stillage.

## Data Availability

Further information and data can be found in Additional file 1. In addition to this, datasets used and/or analysed during the current study are available from the corresponding author on reasonable request.

## References

[CR1] Alyassin M (2019) Arabinoxylan prebiotic co-production within integrated biorefineries. Doctoral thesis, University of Huddersfield

[CR2] Anderson C, Simsek S (2019). A novel combination of methods for the extraction and purification of arabinoxylan from byproducts of the cereal industry. Food Measure.

[CR3] Barros CP, Silva R, Guimarães JT, Balhtazar CF, Verruck S, Pimentel TC, Esmerino EA, Freitas MQ, Duarte MCK, Silva MC, Da Cruz AG, Chhikara N, Panghal A, Chaudhary G (2022). Prebiotics and synbiotics in functional foods. Functional foods.

[CR4] Belitz H-D, Grosch W, Schieberle P (2009). Food chemistry.

[CR5] Chatzifragkou A, Charalampopoulos D (2018). Distiller’s dried grains with solubles (DDGS) and intermediate products as starting materials in biorefinery strategies. Sustainable recovery and reutilization of cereal processing by-products.

[CR6] Chatzifragkou A, Kosik O, Prabhakumari PC, Lovegrove A, Frazier RA, Shewry PR, Charalampopoulos D (2015). Biorefinery strategies for upgrading distillers’ dried grains with solubles (DDGS). Process Biochem.

[CR7] Flodman HR, Boyer EJ, Muthukumarappan A, Noureddini H (2012). Extraction of soluble fiber from distillers’ grains. Appl Biochem Biotechnol.

[CR8] Kaltschmitt M, Hartmann H, Hofbauer H (2016). Energie aus Biomasse.

[CR9] Kehili M, Schmidt LM, Reynolds W, Zammel A, Zetzl C, Smirnova I, Allouche N, Sayadi S (2016). Biorefinery cascade processing for creating added value on tomato industrial by-products from Tunisia. Biotechnol Biofuels.

[CR10] Kim Y, Mosier NS, Hendrickson R, Ezeji T, Blaschek H, Dien B, Cotta M, Dale B, Ladisch MR (2008). Composition of corn dry-grind ethanol by-products: DDGS, wet cake, and thin stillage. Bioresour Technol.

[CR11] Kosik O, Powers SJ, Chatzifragkou A, Prabhakumari PC, Charalampopoulos D, Hess L, Brosnan J, Shewry PR, Lovegrove A (2017). Changes in the arabinoxylan fraction of wheat grain during alcohol production. Food Chem.

[CR12] Lamp A (2020) Proteingewinnung aus Bioethanolschlempe. Dissertation, Technische Universität Hamburg

[CR13] McIlvaine TC (1921). A buffer solution for colorimetric comparison. J Biol Chem.

[CR14] Misailidis N, Campbell GM, Du C, Sadhukhan J, Mustafa M, Mateos-Salvador F, Weightman RM (2009). Evaluating the feasibility of commercial arabinoxylan production in the context of a wheat biorefinery principally producing ethanol. Chem Eng Res Des.

[CR15] Pedersen MB, Dalsgaard S, Knudsen KB, Yu S, Lærke HN (2014). Compositional profile and variation of distillers dried grains with solubles from various origins with focus on non-starch polysaccharides. Anim Feed Sci Technol.

[CR16] Pereira LMS, Milan TM, Tapia-Blácido DR (2021). Using Response surface methodology (RSM) to optimize 2G bioethanol production: a review. Biomass Bioenergy.

[CR17] Roberfroid M (2007). Prebiotics: the concept revisited. J Nutr.

[CR18] Roth M, Jekle M, Becker T (2019). Opportunities for upcycling cereal byproducts with special focus on Distiller's grains. Trends Food Sci Technol.

[CR19] Ruiz HA, Conrad M, Sun S-N, Sanchez A, Rocha GJM, Romaní A, Castro E, Torres A, Rodríguez-Jasso RM, Andrade LP, Smirnova I, Sun R-C, Meyer AS (2020). Engineering aspects of hydrothermal pretreatment: from batch to continuous operation, scale-up and pilot reactor under biorefinery concept. Bioresour Technol.

[CR20] Singh RD, Banerjee J, Arora A (2015). Prebiotic potential of oligosaccharides: a focus on xylan derived oligosaccharides. Bioactive Carbohydr Diet Fibre.

[CR25] Sluiter A, Hames B, Ruiz R, Scarlata C, Sluiter J, Templeton D (2006) Determination of Sugars, Byproducts, and Degradation Products in Liquid Fraction Process Samples. NREL (LAP)

[CR24] Sluiter A, Hames B, Ruiz R, Scarlata C, Sluiter J, Templeton D, Crocker D (2008) Determination of Structural Carbohydrates and Lignin in Biomass. NREL (LAP)

[CR21] Swennen K, Courtin C, Vanderbruggen B, Vandecasteele C, Declour J (2005). Ultrafiltration and ethanol precipitation for isolation of arabinoxylooligosaccharides with different structures. Carbohydr Polym.

[CR22] Zerback T, Schumacher B, Weinrich S, Hülsemann B, Nelles M (2022). Hydrothermal pretreatment of wheat straw—evaluating the effect of substrate disintegration on the digestibility in anaerobic digestion. Processes.

[CR23] Zimmermann A, Visscher C, Kaltschmitt M (2021). Plant-based fructans for increased animal welfare: provision processes and remaining challenges. Biomass Conv Bioref.

