# Supplementary material for: Process options for the recovery of a pentosan-enriched fraction from wheat-based bioethanol thin stillage
Source: Bioresour Bioprocess. 2023 Sep 2;10(1):59. doi: 10.1186/s40643-023-00679-8 (PMC10991448; doi:10.1186/s40643-023-00679-8)
Supplement: Supplementary file 1 — Additional file 1. Additional material - Process options for the recovery of a pentosan-enriched fraction from wheat-based bioethanol thin stillage. [file 40643_2023_679_MOESM1_ESM.docx]

Additional material - Process options for the recovery of a pentosan-enriched fraction from wheat-based bioethanol thin stillage

Zimmermann, A.; Scherzinger, M.; Kaltschmitt, M.

**Enzymatic treatment**

Table S1 shows the results for the determination of the enzymatic optimum of AB Enzymes ROHALASE® VISCO-SEP for thin stillage’s solids (20 min reaction time, 20 μL enzyme solution, 20 mL buffer and 0.5 g solid). Based hereon, the optimum conditions for an enzymatic treatment are assessed to be 44 °C and pH 4.8 (Figure S1).Table S2 and Table S3 show the corresponding ANOVA tables.

Table S1 Central composite design from DesignExpert® for the enzymatic treatment of thin stillage’s solids using ROHALASE® VISCO-SEP. Factors, corresponding values and responses

| Run | Factor A | Factor B | Response 1 | Response 2 |
| --- | --- | --- | --- | --- |
| - | Temperature (°C) | pH value | Pentosan content (%DM) | Pentosan yield (%) |
| 1 | 60 | 5.5 | 5.73 | 8.7 |
| 2 | 60 | 5.5 | 5.65 | 11.1 |
| 3 | 88 | 5.5 | 5.86 | 6.6 |
| 4 | 60 | 5.5 | 5.68 | 9.9 |
| 5 | 60 | 7.6 | 6.12 | 0.1 |
| 6 | 32 | 5.5 | 5.68 | 10.3 |
| 7 | 60 | 5.5 | 5.75 | 8.8 |
| 8 | 80 | 4 | 5.76 | 8.4 |
| 9 | 80 | 7 | 6.12 | 0.6 |
| 10 | 60 | 3.4 | 5.72 | 9.1 |
| 11 | 40 | 7 | 5.87 | 6 |
| 12 | 40 | 4 | 5.71 | 9.8 |
| 13 | 60 | 5.5 | 5.73 | 8.5 |

Figure S1 Result of the numerical optimisation using DesignExpert®. Goal: Minimising the pentosan content in the solid while maximising the pentosan yield (desirability = 0.980)

Table S2 ANOVA for pentosan content model

| **Source** | **Sum of Squares** | | **df** | **Mean square** | | **F-value** | **p-value** | |  |
| --- | --- | --- | --- | --- | --- | --- | --- | --- | --- |
| Model | 0.2850 | | 5 | 0.0570 | | 49.78 | < 0.0001 | | significant |
| A-Temperature | 0.0385 | | 1 | 0.0385 | | 33.60 | 0.0007 | |  |
| B-pH | 0.1473 | | 1 | 0.1473 | | 128.63 | < 0.0001 | |  |
| AB | 0.0100 | | 1 | 0.0100 | | 8.73 | 0.0212 | |  |
| A² | 0.0088 | | 1 | 0.0088 | | 7.69 | 0.0276 | |  |
| B² | 0.0857 | | 1 | 0.0857 | | 74.87 | < 0.0001 | |  |
| **Residual** | 0.0080 | | 7 | 0.0011 | |  |  | |  |
| Lack of Fit | 0.0011 | | 3 | 0.0004 | | 0.2199 | 0.8782 | | not significant |
| Pure Error | 0.0069 | | 4 | 0.0017 | |  |  | |  |
| **Cor Total** | 0.2930 | | 12 |  | |  |  | |  |
| **Std. Dev.** | | 0.0338 | | | **R²** | | | 0.9726 | |
| **Mean** | | 5.80 | | | **Adjusted R²** | | | 0.9531 | |
| **C.V. %** | | 0.5835 | | | **Predicted R²** | | | 0.9358 | |
|  | |  | | | **Adeq. Precision** | | | 19.0749 | |

Table S3 ANOVA for pentosan yield model

| **Source** | **Sum of Squares** | | **df** | **Mean square** | | **F-value** | **p-value** | |  |
| --- | --- | --- | --- | --- | --- | --- | --- | --- | --- |
| Model | 155.37 | | 9 | 17.26 | | 17.34 | < 0.0001 | | significant |
| A-Temperatur | 103.59 | | 1 | 103.59 | | 104.08 | < 0.0001 | |  |
| B-Zeit | 34.50 | | 1 | 34.50 | | 34.66 | < 0.0001 | |  |
| C-c(NaOH) | 12.74 | | 1 | 12.74 | | 12.80 | 0.0030 | |  |
| AB | 0.6591 | | 1 | 0.6591 | | 0.6622 | 0.4294 | |  |
| AC | 0.0207 | | 1 | 0.0207 | | 0.0208 | 0.8873 | |  |
| BC | 0.0025 | | 1 | 0.0025 | | 0.0025 | 0.9611 | |  |
| A² | 4.32 | | 1 | 4.32 | | 4.34 | 0.0561 | |  |
| B² | 11.55 | | 1 | 11.55 | | 11.60 | 0.0043 | |  |
| C² | 1.66 | | 1 | 1.66 | | 1.67 | 0.2169 | |  |
| **Residual** | 13.93 | | 14 | 0.9953 | |  |  | |  |
| Lack of Fit | 10.06 | | 7 | 1.44 | | 2.60 | 0.1153 | | not significant |
| Pure Error | 3.87 | | 7 | 0.5530 | |  |  | |  |
| **Cor Total** | 169.30 | | 23 |  | |  |  | |  |
| **Std. Dev.** | | 0.9977 | | | **R²** | | | 0.9177 | |
| **Mean** | | 10.77 | | | **Adjusted R²** | | | 0.8648 | |
| **C.V. %** | | 9.26 | | | **Predicted R²** | | | 0.6781 | |
|  | |  | | | **Adeq. Precision** | | | 15.2765 | |

**Alkaline treatment**

Table S4 shows the data of the alkaline treatment using design of experiment. Table S5 and Table S6 show the Analysis of Variance (ANOVA) for the corresponding models of the alkaline treatment of thin stillage’s solid phase. Evaluation was done with the help of DesignExpert® (Stat-Ease).

Table S4 Advanced central composite design from DesignExpert® for the alkaline treatment of thin stillage’s solids: Factors, corresponding values and responses

| Run | Factor A | Factor B | Factor C | Response 1 | Response 2 |
| --- | --- | --- | --- | --- | --- |
| - | Temperature (°C) | Reaction time (min) | NaOH concentration (mol/L) | Pentosan yield (%) | Pentosan content solid phase (%DM) |
| 1 | 40 | 60 | 0.1 | 61.5 | 5.94 |
| 2 | 60 | 221 | 0.15 | 77.8 | 13.274 |
| 3 | 80 | 180 | 0.1 | 76.1 | 12.527 |
| 4 | 80 | 180 | 0.2 | 81.2 | 14.85 |
| 5 | 60 | 120 | 0.15 | 70.5 | 12.645 |
| 6 | 40 | 180 | 0.2 | - | 9.538 |
| 7 | 60 | 120 | 0.15 | 70.3 | 11.731 |
| 8 | 60 | 120 | 0.23 | 76.6 | 12.472 |
| 9 | 80 | 60 | 0.2 | 65.9 | 13.029 |
| 10 | 60 | 120 | 0.15 | 72.9 | 12.885 |
| 11 | 94 | 120 | 0.15 | 81.3 | 14.811 |
| 12 | 40 | 180 | 0.1 | 56.4 | 8.896 |
| 13 | 40 | 60 | 0.2 | - | 8.518 |
| 14 | 60 | 120 | 0.07 | 66.8 | 8.96 |
| 15 | 60 | 19 | 0.15 | 68.1 | 5.614 |
| 16 | 26 | 120 | 0.15 | 71.2 | 5.487 |
| 17 | 60 | 120 | 0.15 | 71 | 12.113 |
| 18 | 60 | 120 | 0.15 | 74 | 10.88 |
| 19 | 80 | 60 | 0.1 | 81.2 | 12.009 |
| 20 | 60 | 120 | 0.15 | 71.5 | 12.686 |
| 21 | 50 | 120 | 0.15 | 69.4 | 10.282 |
| 22 | 70 | 120 | 0.15 | 74.2 | - |
| 23 | 40 | 180 | 0.2 | - | 10.721 |
| 24 | 40 | 60 | 0.1 | 67.3 | - |
| 25 | 60 | 19 | 0.15 | 61.6 | 7.395 |
| 26 | 80 | 60 | 0.1 | 75 | 11.223 |

Table S5 ANOVA for pentosan yield model

| **Source** | **Sum of Squares** | | **df** | **Mean square** | | **F-value** | **p-value** | |  |
| --- | --- | --- | --- | --- | --- | --- | --- | --- | --- |
| Model | 830.51 | | 9 | 92.28 | | 14.30 | < 0.0001 | | significant |
| A-Temperature | 93.08 | | 1 | 93.08 | | 14.43 | 0.0022 | |  |
| B-Reaction time | 114.63 | | 1 | 114.63 | | 17.77 | 0.0010 | |  |
| C-c(NaOH) | 106.73 | | 1 | 106.73 | | 16.54 | 0.0013 | |  |
| AB | 6.42 | | 1 | 6.42 | | 0.9954 | 0.3366 | |  |
| AC | 140.57 | | 1 | 140.57 | | 21.79 | 0.0004 | |  |
| BC | 143.54 | | 1 | 143.54 | | 22.25 | 0.0004 | |  |
| A² | 30.31 | | 1 | 30.31 | | 4.70 | 0.0493 | |  |
| B² | 2.59 | | 1 | 2.59 | | 0.4022 | 0.5369 | |  |
| C² | 0.3083 | | 1 | 0.3083 | | 0.0478 | 0.8304 | |  |
| **Residual** | 83.86 | | 13 | 6.45 | |  |  | |  |
| Lack of Fit | 37.16 | | 6 | 6.19 | | 0.9284 | 0.5280 | | not significant |
| Pure Error | 46.70 | | 7 | 6.67 | |  |  | |  |
| **Cor Total** | 914.37 | | 22 |  | |  |  | |  |
| **Std. Dev.** | | 2.54 | | | **R²** | | | 0.9083 | |
| **Mean** | | 71.38 | | | **Adjusted R²** | | | 0.8448 | |
| **C.V. %** | | 3.56 | | | **Predicted R²** | | | 0.6226 | |
|  | |  | | | **Adeq. Precision** | | | 14.6125 | |

Table S6 ANOVA for pentosan content model

| **Source** | **Sum of Squares** | | **df** | **Mean square** | | **F-value** | **p-value** | |  |
| --- | --- | --- | --- | --- | --- | --- | --- | --- | --- |
| Model | 155.37 | | 9 | 17.26 | | 17.34 | < 0.0001 | | significant |
| A-Temperature | 103.59 | | 1 | 103.59 | | 104.08 | < 0.0001 | |  |
| B-Reaction time | 34.50 | | 1 | 34.50 | | 34.66 | < 0.0001 | |  |
| C-c(NaOH) | 12.74 | | 1 | 12.74 | | 12.80 | 0.0030 | |  |
| AB | 0.6591 | | 1 | 0.6591 | | 0.6622 | 0.4294 | |  |
| AC | 0.0207 | | 1 | 0.0207 | | 0.0208 | 0.8873 | |  |
| BC | 0.0025 | | 1 | 0.0025 | | 0.0025 | 0.9611 | |  |
| A² | 4.32 | | 1 | 4.32 | | 4.34 | 0.0561 | |  |
| B² | 11.55 | | 1 | 11.55 | | 11.60 | 0.0043 | |  |
| C² | 1.66 | | 1 | 1.66 | | 1.67 | 0.2169 | |  |
| **Residual** | 13.93 | | 14 | 0.9953 | |  |  | |  |
| Lack of Fit | 10.06 | | 7 | 1.44 | | 2.60 | 0.1153 | | not significant |
| Pure Error | 3.87 | | 7 | 0.5530 | |  |  | |  |
| **Cor Total** | 169.30 | | 23 |  | |  |  | |  |
| **Std. Dev.** | | 0.9977 | | | **R²** | | | 0.9177 | |
| **Mean** | | 10.77 | | | **Adjusted R²** | | | 0.8648 | |
| **C.V. %** | | 9.26 | | | **Predicted R²** | | | 0.6781 | |
|  | |  | | | **Adeq. Precision** | | | 15.2765 | |
